# Nutrient content of sorghum hybrid lines between Gadam and hard coat tannin sorghum cultivars

**DOI:** 10.1002/fsn3.2830

**Published:** 2022-03-21

**Authors:** Cecilia A. Shinda, Paul N. Nthakanio, Josiah N. Gitari, Steven Runo, Simon Mukono, Samuel Maina

**Affiliations:** ^1^ Department of Water and Agricultural Resource Management University of Embu Embu Kenya; ^2^ Department of Biochemistry, Microbiology and Biotechnology Kenyatta University Nairobi Kenya; ^3^ Department of Physical Sciences University of Embu Embu Kenya; ^4^ Department of Biological Sciences University of Embu Embu Kenya

**Keywords:** crude protein, F_1_, gadam, nutrient content, tannin

## Abstract

Sorghum is an important food crop in the world that exhibits a predominant role in fulfilling the nutritional requirements, particularly in low‐income group populations of marginal areas in Kenya. It is a principal source of proteins, carbohydrates, fats, and crude fibers (CFs), which are important nutrients necessary for human development and health. Reduced tannin in sorghum grains is desirable since it affects the availability of nutrients. This study aimed at assessing the nutrient content in filial generation one (F_1_) developed between Gadam (sorghum), which is low in tannin and hard coat tannin (sorghum) cultivars. The nutrient content analyses were carried out from samples collected in a completely randomized design experiment. Crude protein (CP) and tannin content were analyzed using the modified Kjeldahl method and vanillin‐HCl methanol method, respectively, whereas moisture, fat, CF, ash, and carbohydrate contents were determined using Association of Official Analytical Chemists methods. Data collected were subjected to analysis of variance using R statistical software. Among the F_1_s, Kari/Mtama‐1 x Gadam recorded the highest CP value of 10.390%. This differed significantly from Gadam x Kari/Mtama‐1 which recorded CP content of 9.770%. Kari/Mtama‐1 x Gadam recorded the highest fat and moisture contents of 2.299% and 8.600%, respectively. The highest CF content of 3.433% was recorded in Gadam x Serena. Gadam x Kari/Mtama‐1 recorded the highest ash content of 1.619%, whereas the highest carbohydrate (84.503%) and tannin content (0.771 mg/g) means were recorded in Seredo x Gadam. Results demonstrated that the choice of maternal and paternal parent influence CP, CF, and carbohydrate contents. Among the F_1_s, tannin content ranged from 0.106 to 0.771 mg/g compared to 0.953 to 1.763 mg/g recorded in Serena and Seredo (hard coat seeded cultivars). This is an indication that tannin can be downregulated through hybridization.

## Graphical abstract

Results demonstrated that the choice of maternal and paternal parent influence crude proteins, crude fiber, and carbohydrate contents. Among the F1s, tannin content ranged from 0.106 to 0.771 mg/g compared to 0.953 to 1.763 mg/g recorded in Serena and Seredo (hard coat seeded cultivars). This is an indication that tannin can be downregulated through hybridization.

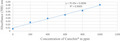

## 1 INTRODUCTION

Sorghum (*Sorghum bicolor* (L.) Moench) is an important source of food and income for many resource‐poor small‐scale farmers, in the world, and in Kenya it is popular with communities living in arid and semiarid areas (Reddy et al., 2010). It is a drought‐tolerant cereal crop, highly resistant to variations in temperatures and soil toxicities as compared to other cereal crops such as maize (Hadebe et al., 2017). Sorghum is a principal source of proteins, carbohydrates, fats, and crude fiber (CF), both of which are important nutrients necessary for human development and health (Duodu et al., 2003; Jakobek, 2015). Its flour has been used in the preparation of porridge, alcoholic beverages, and flat bread (Kahlon & Chiu, 2012; Taylor & Duodu, 2019; Vange et al., 2014). Delicacies and flat snacks are also produced from sorghum grains (Nirmal et al., 2017; Zabala et al., 2015). Sorghum's nutritional value depends on the growing conditions and variety (Sowiński & Liszka‐Brandau, 2019).

Mature sorghum kernel is composed of the embryo (10%), endosperm (80%), and pericarp (8%) (Rooney et al., 2012), but the relative proportions may vary with genetic background, degree of maturity, and growth environment (Rooney et al., 2014). Sorghum pericarp is majorly composed of carotenoids, non‐starch polysaccharides, and phenolic compounds including tannins, 3‐deoxyanthocyanidins, and phenolic acids (Moraiscardoso et al., 2015). The embryo is rich in minerals, proteins, B‐complex vitamin, fat, soluble vitamins, and lipids; thus, removal of the outer pericarp increases the protein and reduces the lipid, cellulose, and mineral content of the grain (Dicko et al., 2006; Etuk et al., 2012). The grain endosperm is composed of minerals, B‐complex vitamins, and starch (Morais Cardoso et al., 2017; Mwenda et al., 2019). Starch (32.1–72.5 g/100g) in sorghum is composed mainly of amylose (3.5%–19.0%) and amylopectin (81.0%–96.5%) (Singh, Dartois, et al., 2010; Udachan et al., 2012). Singh, Sodhi, et al. (2010) reported that the proportion of amylose and amylopectin influences the rheological and digestibility properties of sorghum.

Sorghum varieties with pigmented testa are rich in phenolic compounds especially tannins (Mwenda et al., 2019). Tannin is a high‐molecular‐weight polyphenol that is able to bond more preferentially to proteins and inhibits many enzymes in in vitro assays, thus reducing the overall food value (Frazier et al., 2010; Hagerman & Butler, 2010). It has been reported to reduce growth rate and feed efficiency in animals (Mitaru et al., 2012). The availability of proteins, minerals, and starch in sorghum is also reduced by the presence of tannins (McIntosh & Vancov, 2010). De Oliveira et al. (2014) documented that tannin in sorghum reduces digestibility and efficiency of utilization of absorbed nutrients from 3% to 15%. Tannin interferes with protein structure and its function, thus lowering its quality (Jakobek, 2015). It changes the protein structure as a result of weak hydrophobic sites on the protein surface which occur when tannins bind to the hydrophobic sites of the protein (Yuksel et al., 2010). This may lead to possible changes in the folding of proteins and their functionality (Jakobek, 2015).

Starch digestibility in sorghum is low compared to other cereals, and this is attributed to strong associations between tannin, starch granules, and proteins (Barros et al., 2012; Mkandawire et al., 2013). Taylor and Emmambux (2010) documented that the soluble fibers (10.0% – 25.0%) and insoluble fibers (75.0% – 90.0%) are the major sources of non‐starch polysaccharides (6.0 – 15.0 g/100 g). Sorghum proteins contain high amounts of nonpolar amino acids like leucine, proline, and alanine (Mesa‐Stonestreet et al., 2010). Sorghum is rich in glutamic acid but limiting in lysine (Mokrane et al., 2010; Moraes et al., 2012). It is known to be the source of fat‐soluble vitamins (D, E and K) and some B‐complex vitamins such as riboflavin, thiamine, and pyridoxine (Morais Cardoso et al., 2017).

Sorghum contains resistant starch (RS) (Dicko et al., 2006). Nutritionally, starch has been classified as RS, slowly digestible starch (SDS), and rapidly digestible starch (RDS) (Amoako & Awika, 2016). SDS has been reported to increase the satiety as it results in a slower sustained postprandial glucose response (Aller et al., 2011). RDS leads to a rapid increment in the level of blood glucose. The fractions of RS have been reported to function as dietary fiber and help in escaping enzyme hydrolysis in the small intestine (Barros et al., 2012, 2014). RS in sorghum has been recommended in fighting human obesity and feeding diabetic people; however, it reduces the digestibility of food, especially for infants (Dicko et al., 2006).

Sorghum varieties with low tannin content grown globally include Mugud, Giza 15, Macia, Gambela‐1170, Teshale, and NES 1007 (Tasie & Gebreyes, 2020; Wedad et al., 2008; Youssef et al., 1988). Methods such as dehulling and fermentation have been used to reduce tannin content in sorghum varieties (Chibber et al., 1978; Khalifa & Tinay, 1994). However, some of these methods are associated with the loss of other nutrients, especially proteins and amino acids, from the grain (Chibber et al., 1978). Sorghum breeders have bred for sorghum varieties with low tannin content such as SH1, SH2, and SH3 for improved food quality (Gurbuz & Davies, 2010). Tannin is genetically controlled by additive alleles (Hill, 2015; Wu et al., 2012). Hybrid inherits only one of the tannin gene pairs, hence lowering the genetic dosage. Since tannin is under quantitative trait loci (QTL), the level of tannin in hybrids is hypothesized to be low compared to parents.

Common sorghum cultivars cultivated in Kenya include Gadam, Kari/Mtama‐1, Serena, and Seredo (Timu et al., 2014). Gadam sorghum is a semidwarf early maturing cultivar with white sweet grains (Kagwiria, 2012). The crop matures in two and a half to three months depending on the rainfall amount and altitude of the area (Bosire, 2019). The average yield of Gadam per hectare is 3.15 tons (Olmstead & Rhode, 2014). Because of its sweet grains, the crop is highly susceptible to bird damage (Este et al., 2019), thus lowering the yield. However, the crop is highly tolerant to drought and hence suitable for cultivation in marginal regions of Kenya including Makueni, Kitui, Tharaka, Mbeere, Mwingi, Kilifi, Machakos, Moyale, Tana river, Kajiado, and Marsabit districts (Karanja et al., 2006). The cultivar is good for home consumption and commercialization (Orr et al., 2013). It is used in the brewing industry to make malted beverages such as beer due to high malting quality (Orr et al., 2013). Kari/Mtama‐1 is a tall cultivar with large cream white grains (Lado & Muthomi, 2020). It takes three and a half to four months to reach physiological maturity and is capable of yielding 3.8 tons per hectare (Karanja et al., 2014). Kari/Mtama‐1 is highly palatable to birds due to its sweet grains which are low in tannin content (Karanja et al., 2006). The cultivar is cultivated in the lower eastern and upper eastern Kenya (Karanja et al., 2014). Serena is a medium maturing cultivar with brown grains which takes three to three and a half months to mature (Mwadalu & Mwangi, 2013). The crop yields 2.25 tons per hectare, and it is capable of resisting birds’ damage because of tannins in the grains (Monyo et al., 2004). It is cultivated in western and eastern regions of Kenya. Seredo is a medium maturing cultivar with dark brown grains that are cultivated in lower eastern and western Kenya (Mwadalu & Mwangi, 2013). It takes three months to reach physiological maturity and is capable of yielding 2.7 tons per hectare (Karanja et al., 2014). The crop has high tannin content in the grains which makes it tolerate birds attack (Este et al., 2019). Serena and Seredo have a relatively higher yield compared to low tannin varieties due to low bird infestation (Nyangeri & James, 1993).

Improving the nutrient availability of sorghum is critical in enhancing food security (Awika, 2019). Low tannin sorghum grains are ideal for human nutrition and animal feed (Palacios et al., 2021). Gadam sorghum has a high food value due to low tannin but is susceptible to birds infestation reducing its yield (Este et al., 2019). Sorghum cultivars such as Seredo and Serena resist birds’ infestation but have less food value due to high tannin levels (E. Omondi et al., 2014). The F_1_ hybrids developed between Gadam and hard coat tannin sorghum cultivars have been hypothesized to have higher nutritional value and are able to resist bird's attack due to relatively low tannin levels. Therefore, the objective of this study was to determine the nutrient content of sorghum hybrid lines developed between Gadam and hard coat tannin sorghum cultivars. This will help in identifying the sorghum hybrid lines with moderate tannin levels and high food value to be used in sorghum seed production program in Kenya.

## 2 MATERIALS AND METHODS

### 2.1 Materials

F_1_ hybrid seeds were first obtained by crossing Gadam sorghum and the three selected sorghum cultivars: Serena, Seredo, and Kari/Mtama‐1 (check parent) sourced from KALRO seed unit at Katumani, Machakos County. The seeds of hybrid lines namely; Gadam x Serena, Gadam x Seredo, Gadam x Kari/Mtama‐1, their reciprocals, and parents were planted at the University of Embu experimental field in a randomized complete block design (RCBD) and replicated three times. The seeds were grown under observation in an open field for three to four months. The experimental field is in upper midland 2 (UM2) and UM3 Agro‐ecological zones (latitude 0°35’25’’ S, longitude 37° 25’31’’ E, and altitude 1463m above sea level). The soils in the area are that of basic volcanic rocks and are characterized as *Humic Nitisols* (Jaetzold et al., 2007). Samples were taken in triplicates from an experiment laid in an RCBD. At maturity, whole mature grains of each hybrid sorghum line and their parents were harvested manually. The seeds were hand‐threshed and then cleaned by winnowing to remove impurities, air‐dried and ground into fine flours using a high‐speed universal disintegrator (FW80‐I) in the laboratory, and sieved using a 0.5‐mesh screen. Fine flour was packed in polythene bags and then preserved at room temperatures till use.

### 2.2 Crude protein analysis

Crude protein (CP) was determined using the modified Kjeldahl method of Cope (1889) (Sarkar & Haldar, 2005). A blank control with all reaction mixtures without the sample was performed parallel to the sample. The nitrogen value was deduced by subtracting the experimental sample value from the experimental blank value. The deduced nitrogen value obtained was then multiplied by 6.25 conversion factor to obtain protein content as follows:

% Crude protein = [(S‐T) x N x 1.4/w] 6.25.

where,

S = blank titration.

T = titration of the sample.

*N* = normality of standard alkali.

w = sample weight in grams.

### 2.3 Fats analysis

Fat content was determined according to AOAC 945:16 (Horwitz, 2000; Tasie & Gebreyes, 2020) with slight modification that included evaporating the major portion of the solvent inside the fume hood. It was later calculated as follows:

% fat = [(W2‐W1)/W] 100.

where,

W2 = weight of the flask and fat deposit.

W1 = weight of the empty flask only.

W = weight of the sample taken for the test.

### 2.4 Crude fiber analysis

The CF was determined as per the method of AOAC 962:09 (Horwitz & Latimer, 2005; FSSAI, 2016). It was later calculated as follows:

% Crude fiber = [(W1‐W2)/W] 100.

where,

W1 = weight in grams of Gooch crucible and contents before ashing.

W2 = weight in grams of Gooch crucible containing ash.

W = weight in grams of the dried material taken for the test.

### 2.5 Ash content analysis

Ash content was determined as per the method of AOAC 923:03 (Horwitz & Latimer, 2005) and then it was computed as:

% Ash = [(W2‐W1)/W] 100.

where,

W2= weight in grams of the crucible with the ash after ignition in the muffle furnace.

W = weight in grams of the sample taken for test.

W1 = weight in grams of the empty clean and dry crucible.

### 2.6 Moisture content analysis

Moisture content was determined from seeds harvested at physiological maturity. It was determined according to AOAC 925:10 (Horwitz, 2000; Tasie & Gebreyes, 2020) with slight modifications that included drying the samples in the drying oven for 2 hr at 105°C. It was then computed as follows:

% Moisture = [(WI‐W2/ W] 100.

where,

W1 = weight in grams of the petri‐dish with sample before drying.

W2 = weight in grams of the petri‐dish with the sample after drying.

W = weight in grams of the sample taken for test.

### 2.7 Total carbohydrate analysis

Total carbohydrates from the sorghum samples were obtained as per Pearson (1976) by subtracting the obtained figure of moisture content, fats, ash content, proteins from 100% as follows:

Total carbohydrate (%) = [100‐(moisture (%) + fats (%) + ash (%) + proteins (%))].

### 2.8 Tannin content analysis

**FIGURE 1:**
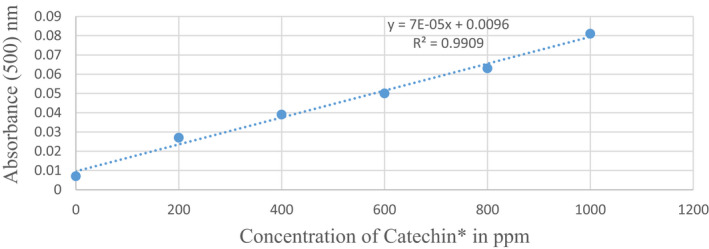
Standard curve on Catechin levels. Nm, nanometer; ppm, parts per million; Catechin^*^ and tannin have the same components

Tannin content was determined using the modified vanillin‐HCl assay method of Price et al. (1978) using a spectrophotometer (model, ME 801). To prepare a standard curve, the absorbance of the colored intensity rate for each concentration of Catechin (ppm) was first measured at 500 nm using a spectrophotometer (Table 1). The slope of the line was determined thereafter using concentration of Catechin (0, 200, 400, 600, 800, and 1000 ppm) as the x‐axis and the absorbance values as the y‐axis in Microsoft Excel (Figure 1). Tannin concentration was calculated using the quadratic equation obtained from the standard calibration curve:

**TABLE 1:** The absorbance of the colored intensity rate for different concentrations of Catechin solution in parts per million

Standards	
Concentration of Catechin^*^ (ppm)	Absorbance (500) nm
0	0.007
200	0.027
400	0.039
600	0.050
800	0.063
1000	0.081

Abbreviation: ppm, parts per million; nm: nanometer.

Y = 7 * 10 ^–5^ x + 0.0096.

where Y = absorbance, X = concentration.

### 2.9 Data analysis

Statistical data for protein content, fat, CF, ash, carbohydrate, moisture, and tannin content was subjected to a one‐way analysis (ANOVA) using R statistical software (R Development Core Team, 2015). Mean separation was done using Tukey's Studentized Range (HSD) at 95% confidence level using package agricolae in R (De Mendibru, 2019). Pearson's correlation was done to compare the degree of association between the traits analyzed.

## 3 RESULTS

### 3.1 Nutrient content of sorghum F_1_ hybrids and their parents

The CP, fat content, CF, ash content, total carbohydrates, moisture, and tannin content of F_1_ hybrids and their parents are shown in Table 2. There was a significant difference (*p* < .001) in CP between F_1_ hybrids and their reciprocals. The parents also differed significantly in CP with Kari/Mtama‐1 recording the highest CP mean of 10.133% while Seredo recording the lowest mean of 5.323%. Gadam and Kari/Mtama‐1 and its reciprocal had the highest CP content among the hybrids; however, they did not differ significantly from Kari/Mtama‐1. It is only Seredo x Gadam (with 1.691%) that had significantly lower fat content compared to other hybrids and also the parents.

**TABLE 2:** Nutritional compositions (g/100g) and tannin content (mg/g) of sorghum F_1_ hybrids and their parents. S.E, standard error

Treatments	Crude protein (%) (Mean ± S.E)	Fat (%) (Mean ± S.E)	Crude fiber (%) (Mean ± S.E)	Ash (%) (Mean ± S.E)	Moisture (%) (Mean ± S.E)	Carbohydrate (%) (Mean ± S.E)	Tannin (mg/g) (Mean ± S.E)
Parents							
Gadam	7.660^e^ ± 0.127	2.050^ab^ ± 0.066	3.180^ab^ ± 0.092	1.318^b^ ± 0.040	7.200^c^ ± 0.100	81.772^c^ ± 0.171	0.653^c^ ± 0.064
Kari/Mtama−1	10.133^ab^ ± 0.073	2.197^ab^ ± 0.046	2.230^e^ ± 0.058	1.215^b^ ± 0.028	9.667^a^ ± 0.333	76.788^f^ ± 0.236	0.034^d^ ± 0.000
Serena	5.900^g^ ± 0.000	1.931^bc^ ± 0.058	3.150^ab^ ± 0.100	1.329^ab^ ± 0.081	5.993^de^ ± 0.007	84.847^a^ ± 0.047	0.953^b^ ± 0.031
Seredo	5.323^h^ ± 0.073	2.114^ab^ ± 0.070	3.520^a^ ± 0.111	1.453^ab^ ± 0.032	5.433^e^ ± 0.088	85.677^a^ ± 0.165	1.763^a^ ± 0.130
Direct crosses							
Gadam x Kari/Mtama−1	9.770^bc^ ± 0.070	2.159^ab^ ± 0.082	2.663^cd^ ± 0.100	1.619^a^ ± 0.056	8.100^b^ ± 0.100	78.352^e^ ± 0.162	0.177^d^ ± 0.000
Gadam x Serena	9.557^cd^ ± 0.073	2.034^ab^ ± 0.046	3.433^a^ ± 0.083	1.227^b^ ± 0.014	7.103^c^ ± 0.103	80.079^de^ ± 0.188	0.281^d^ ± 0.041
Gadam x Seredo	9.227^d^ ± 0.037	2.116^ab^ ± 0.034	3.197^ab^ ± 0.075	1.352^ab^ ± 0.084	6.427^d^ ± 0.073	80.878^d^ ± 0.152	0.582^c^ ± 0.029
Reciprocal crosses							
Kari/Mtama−1 x Gadam	10.390^a^ ± 0.110	2.299^a^ ± 0.104	2.493^de^ ± 0.063	1.333^ab^ ± 0.071	8.600^b^ ± 0.100	77.378^f^ ± 0.244	0.106^d^ ± 0.036
Serena x Gadam	6.443^f^ ± 0.064	2.167^ab^ ± 0.028	2.953^bc^ ± 0.055	1.349^ab^ ± 0.058	6.413^d^ ± 0.049	83.628^b^ ± 0.087	0.696^bc^ ± 0.081
Seredo x Gadam	6.343^f^ ± 0.124	1.691^c^ ± 0.087	2.280^de^ ± 0.087	1.360^ab^ ± 0.070	6.103^d^ ± 0.058	84.503^b^ ± 0.188	0.771^bc^ ± 0.034
*p*‐value	<.001^***^	<.001^***^	<.001^***^	<.001^***^	.005^**^	<.001^***^	<.001^***^

Note

Means with the same letter within the column are not significantly different. ^**^ Significant at 1%; ^***^significant at 0.1%. mg/g: milligram per gram; %: percentage.

Among the hybrids, Gadam x Kari/Mtama‐1 had no significant difference with its reciprocal in CF; however, significant differences were observed in CF among other hybrids compared to their reciprocals. Among the parents, Kari/Mtama‐1 (which had the highest protein content among the parents) recorded the lowest CF content. Seredo had the highest CF content; however, among its hybrid with Gadam, it only differed significantly with Seredo x Gadam. Hybrids did not differ significantly in ash content apart from Gadam x Kari/Mtama‐1 and Gadam X Serena. Kari/Mtama‐1 x Gadam had the highest moisture content mean of 8.600 though not significantly different from Gadam x Kari/Mtama‐1.

The F_1_ hybrids Seredo x Gadam and Serena x Gadam had the highest carbohydrate content that was significantly higher than Gadam x Seredo and Gadam x Serena. Carbohydrate in hybrids between Gadam x Seredo, Gadam x Serena and their reciprocals was significantly lower compared to Serena and Seredo parents. Gadam had a higher carbohydrate content compared to Gadam x Kari/Mtama‐1 and Kari/Mtama‐1 x Gadam. Tannin content varied significantly (*p* < .001) among the parents with Seredo recording the highest mean followed by Serena, Gadam, and Kari/Mtama‐1 (Shinda et al., 2021). For parents with high tannin levels, their hybrids recorded significantly lower tannin levels apart from Serena x Gadam whose difference was not significant compared to parental Serena.

Pearson correlations of nutrient content of sorghum are shown in Table 3. A significant positive correlation was observed between CP and moisture content (*r* = 0.798, *p* < .057) and between tannin content and carbohydrates (*r* = 0.965, *p* < .002). Tannin content correlated negatively with CP (*r* = −0.906, *p* < .013) and moisture content (*r* = −0.948, *p* < .004). A negative correlation was also observed between CP and carbohydrates (*r* = −0.964, *p* < .002) and between carbohydrate and moisture content (*r* = −0.924, *p* < .008).

**TABLE 3:** Pearson correlation of proximate compositions of sorghum

Variables	CP	F	CF	A	M	CHO
F	0.627					
CF	0.254	0.271				
A	0.126	0.145	−0.419			
M	**0.798**	0.673	−0.205	0.347		
CHO	**−0.964**	−0.715	−0.086	−0.260	**−0.924**	
T	**−0.906**	−0.638	−0.061	−0.225	**−0.948**	**0.965**

Note

Values in bold are different from 0 with a significance of *p* = .05. CP, crude protein; F, fat; CF, crude fiber; A, ash; M, moisture; CHO, carbohydrates; T, tannin.

## 4 DISCUSSION

Sorghum is a major source of proteins, carbohydrates, fats, and CF, necessary for human development and health (Duodu et al., 2003; Jakobek, 2015). Determining and understanding the nutritional and antinutritional properties of sorghum hybrid lines would aid in the selection of lines with moderate tannin levels with high food value to be used in sorghum hybrid seed production programs. In the current study, significant variations (*p* < .001) were observed in CP content among the parents and also between the F_1_ hybrids (Table 2). The variability in protein content among the parents has been attributed to the genetic make‐up of the parents as well as their macromolecules composition, especially the tannins (Tasie & Gebreyes, 2020). The findings of this study on protein content are in agreement with those of Badigannavar et al. (2016), Jambunathan et al. (1981), and Pontieri et al. (2012) who reported protein content varying from 5.25% to 14.53%, 4.4 to 21.1%, and 7.44 to 9.66%, respectively. Other researchers have documented sorghum protein content range of 10.3% to 14.9% (Johnson et al., 2010), 9.06% to 18.58% (Hamad, 2006), 11.23% to 13.42% (Chung et al., 2011), and 9.06% to 18.58% (Gu‐Ayebeafo Okrah, 2008). Differences in the amount of protein content among the studies are due to the differences in the genotype and environment (Deosthale et al., 1972). Significance difference in CP trait was observed between F_1_ and their reciprocal (Table 2). Thus, the choice of maternal and paternal parents influenced protein content. Kari/Mtama‐1 had the highest CP content among parents. Among the F_1_ hybrids, the highest CP content was realized when Kari/Mtama‐1 was the female parent. This is an indication that CP content can be influenced by cytoplasmic effects. Maternal influence in reciprocals has been reported for protein content in maize (Pollmer et al., 1979).

The fat content ranged from 1.691% in Seredo x Gadam to 2.299% in Kari/Mtama‐1 x Gadam. Results in this study agree with the works of Okoh et al. (1982) who reported that fat content ranged from 1.38 to 3.70. However, higer content value, ranging from 3.44 to 4.90%, have been reported by Buffo et al. (1998) while working on the proximate analysis of sorghum varieties. Seredo x Gadam was the only F_1_ hybrid line that had a significant difference in fat content. All other F_1_s had no significant difference from their parents. This shows that crossbreeding did not bring about significant differences in this trait. Seredo and Gadam parents had the highest CF content. Gadam x Seredo had no significant difference with the parents while Seredo and Gadam recorded a significant difference. Also, Gadam x Serena had a significant difference with its reciprocal but not with the two parents. Thus, the choice of which is maternal or paternal may be a major item in breeding.

CF is a major portion of carbohydrates that cannot easily be digested. Among the F_1_ hybrids, the highest CF value of 3.433% was recorded in Gadam x Serena while the lowest value of 2.493% was recorded in Kari/Mtama‐1 x Gadam. CF content varying from 1.0% to 3.4% (Jambunathan et al., 1981) and 2.166% to 8.587% (Tasie & Gebreyes, 2020) have been reported. The differences in CF in this study and other studies could be attributed to the environment where the crop was grown as well as the type of genotype or the method used in the analysis (Ref). CF is capable of holding oil and water (Elleuch et al., 2011); thus, varieties with high CF content can be useful in yield enhancement and also in making products that need hydration. However, these varieties have low food value since high CF binds minerals together reducing their efficiency for absorption and sometimes leading to minerals deficiency as well as imbalances (Oliveira et al., 2009). The CF contents recorded in this study were within the limits recommended by codex standards (5%) for complementary foods. Thus, both the parents and hybrid sorghum composite flours are suitable for use in home‐based complementary feeding of children aged 6–59 months (Tumuhimbise et al., 2019).

Ash content indicates the total amount of mineral content found in a sample (Jimoh & Abdullahi, 2017). Lines with higher CP content displayed higher ash content but the difference was not significant among the hybrids apart from Gadam x Kari/Mtama‐1 and Gadam x Serena that had a significant difference (Table 2). This indicates that CP and ash content in sorghum can be improved simultaneously. Among the F_1_ hybrids, Gadam x Kari/Mtama‐1 recorded the highest ash content value of 1.619% while the lowest ash content value of 1.227% was recorded in Gadam x Serena. The differences in ash content among the F_1_ hybrid sorghum could be attributed to the genotype as well as the amount and nature of ions available in the soil where the plant is growing (Akinsola, 1993). Different values for ash content among sorghum varieties has been reported by various researchers; for instance: 1.90% to 1.97% (Gassem & Osman, 2003), 1.43% to 1.61% (Chung et al., 2011), 1.01% to 1.56% (Abu et al., 2001), 0.80% to 2.50% (Moharram & Youssef, 1995), and 0.99% to 1.71% (Pontieri et al., 2012).

In cereals, moisture content of less than 15% ensures long‐term storage of the grains without loss of quality or viability that might occur as a result of molding caused by high moisture content (Onimawo et al., 2003). In this study, Kari/Matam‐1 x Gadam recorded the highest moisture content of 8.600% while Seredo x Gadam recorded the lowest moisture content of 6.103%. This suggests that moisture content in sorghum can be ascribed to genotype. Significantly lower carbohydrate content recorded in Gadam x Seredo, Gadam x Serena and their reciprocals compared to Serena and Seredo parents indicate that hybridization lowered carbohydrate content in the F_1_ hybrids. All hybrids and their reciprocals had a significant difference in carbohydrate content. Out of them, Seredo x Gadam recorded carbohydrates content significantly different from all F_1_ hybrids except from Serena x Gadam. The choice of male or female parent was found to influence the levels of carbohydrates for the materials under study.

The F_1_s Gadam x Kari/Mtama‐1, Gadam x Serena, and Kari/Mtama‐1 x Gadam recorded significantly (*p* < .001) lower tannin content compared to the standard line, Gadam (Table 2). This is an indication that tannin can be downregulated through hybridization. Tannin is one of the major antinutritional factors in sorghum (Hariprasanna et al., 2015). It has been reported to bind proteins together and inhibit many enzymes in in vitro assays reducing their efficiency of utilization and digestion (Emmambux & Taylor, 2003; Frazier et al., 2010). Besides, it makes the sorghum grains remain bitter, thus reducing the taste of many food products (Coelho et al., 2007; Tasie & Gebreyes, 2020). Results on tannin content of the parents differ from the findings of Omondi et al. (2012) who reported tannin levels of 0.81% C.E, 0.03% C.E, 2.22% C.E, and 1.2% C.E in Gadam, Kari/Mtama‐1, Seredo, and Serena, respectively. The variations observed between the results could be due to differences in the method used in the analysis (Ref). However, the tannin content range of 0.106 mg/g to 0.771 mg/g observed among the F_1_ hybrids is within the findings by Moharram and Youssef (1995), who reported sorghum tannin content range of 0.02 g/100g to 2.69 g/100g.

A significant positive correlation observed between CP and moisture content indicates no marked barriers to the simultaneous improvement of these traits in commercial hybrid sorghums. Reverse relation between CP and tannin content shows that selection for high CP in sorghum cultivars with low tannin content can easily be realized. Reduced tannin in hybrid grains is desirable since it affects protein availability. Sorghum proteins are less digestible compared to those of other cereal crops like maize (Xiong et al., 2019). This poor digestibility is due to phenolic compounds mainly tannins that are found in most sorghum varieties (Duodu et al., 2003). Tannins have been thought to interact with proteins through hydrophobic interactions and hydrogen bonding, and it has been reported to bind and precipitate most proteins, at least 12 times their own weight of proteins (Butler et al., 2011; Jakobek, 2015).

## 5 CONCLUSION

Choice of maternal and paternal parent influences CPs, CF, and carbohydrates. Tannin is highly influenced by hybridization. This was demonstrated by significantly lower tannin content recorded by all the F_1_ hybrids compared to hard coat sorghum cultivars. In sorghum, high CP and low tannin content can be bred simultaneously since the two traits are negatively correlated. Carbohydrates, CF, and tannins correlate positively in grain sorghum. Based on these findings, there is a need to consider maternal and paternal parents when breeding for CP, CF, and carbohydrates. Sorghum with high protein content and low tannin content can be bred to solve the problem of protein malnutrition among the poor population particularly in Africa, and correlation between variables can aid selection.

## CONFLICT OF INTEREST

No potential conflict of interest has been declared concerning the publication of this paper.

## AUTHOR CONTRIBUTIONS

Cecilia A. Shinda was involved in sample collection from the farm, laboratory data acquisition, and preparation of the first draft of the manuscript. Paul N. Nthakanio, Josiah N. Gitari, Steven Runo, and Simon Mukono gave a lead on the nutrients analysis and were also involved in resource acquisition and supervision. Samuel Maina was involved in data analysis. All authors took part in manuscript preparation and approved the submission of the manuscript.

## Data Availability

The data that support the findings of this study are available from the corresponding author upon reasonable request.
